# Transmembrane proteins in grape immunity: current knowledge and methodological advances

**DOI:** 10.3389/fpls.2024.1515163

**Published:** 2024-12-20

**Authors:** Alessia Gallucci, Deborah Giordano, Angelo Facchiano, Clizia Villano, Domenico Carputo, Riccardo Aversano

**Affiliations:** ^1^ Department of Agricultural Sciences, University of Naples Federico II, Portici, Italy; ^2^ Institute of Food Science, National Research Council, Avellino, Italy

**Keywords:** *Vitis*, receptor, LysM-RLKs, SWEETs, RBOH, NMR, FRET, topology

## Abstract

Transmembrane proteins (TMPs) are pivotal components of plant defence mechanisms, serving as essential mediators in the response to biotic stresses. These proteins are among the most complex and diverse within plant cells, making their study challenging. In spite of this, relatively few studies have focused on the investigation and characterization of TMPs in plants. This is particularly true for grapevine. This review aims to provide a comprehensive overview of TMP-encoding genes involved in grapevine immunity. These genes include Lysin Motif Receptor-Like Kinases (LysM-RLKs), which are involved in the recognition of pathogens at the apoplastic level, Plant Respiratory Burst Oxidase Homologs (Rbohs), which generate reactive oxygen species (ROS) for host defense, and Sugars Will Eventually be Exported Transporters (SWEETs), which play a role in nutrient allocation and stress responses. Furthermore, the review discusses the methodologies employed to study TMPs, including *in vivo*, *in vitro* and in silico approaches, highlighting their strengths and limitations. *In vivo* studies include the assessment of TMP function in whole plants or plant tissues, while *in vitro* experiments focus on isolating and characterizing either specific TMPs or their components. In silico analyses utilize computational tools to predict protein structure, function, and interactions. By identifying and characterizing genes encoding TMPs involved in grapevine immunity, researchers can develop strategies to enhance grapevine resilience and lead to more sustainable viticulture.

## 1 Introduction

Transmembrane proteins (TMPs) are integral components of lipid bilayer membranes in living organisms that function as first barriers to the outside world. They constitute half of the components in cell membranes (Goossens and De Winter, 2018) and are among the most complex and diverse proteins within plant cells, making them difficult to study (Corradi et al., 2019; Barrera et al., 2019). From a structural point of view, TMP differ from other membrane proteins in their folding, which allocates them across the membrane. The protein can cross the membrane with a single passage (bitopic) or a multiple passage (polytopic). The segments that cross the membrane are folded with the typical alpha-helix structure, with the peculiarity of having the side chains of amino acids in contact with the lipid portion of the membrane. Another structural organization of the transmembrane portion, although less frequent, is the beta-barrel type architecture, observed in mitochondria, chloroplasts, and Gram-negative bacteria. In any case, the hydrophobicity of the amino acid side chain is an expected feature in the transmembrane regions, related to the need to adapt to the apolar environment of the lipid region of the membrane. Transmembrane regions are alternated in the amino acid sequence by polar regions that remain in the polar environment outside the membrane, and function as simple connection loops as independent domains with a fold like water-soluble proteins. The alternation of polar and apolar regions is a typical feature that may allow integral membrane proteins to be recognized by the analysis of chemical-physical properties along the amino acid sequence. Typical signal peptides can also be recognized. The mechanism of the embedding in the lipid membrane is the object of many studies and is still poorly understood (Argudo, 2024).

In plants, TMPs take over numerous critical functions like solute transport (active/passive) (Pellizzaro et al., 2015), signal transduction (Kemmerling et al., 2011; Mohd-Radzman et al., 2015; Song et al., 2015; Imin et al., 2018) and cell-cell recognition (Langhans et al., 2017). Because of these functions, they participate in many physiological and pathological processes such as growth, development (Clark et al., 1997; van der Knaap et al., 1999; Osakabe et al., 2005; ten Hove et al., 2011) and photosynthesis (Liu and Last, 2015; Okumura et al., 2016). TMPs can also respond to environmental stresses by triggering physiological adaptation that enhance plant resilience (Kraffe et al., 2007). Over recent years, researcher has focused on elucidating TMPs roles in plant immunity, particularly in pathogen detection, signal transduction, and defense activation (Gull et al., 2019). Extensive studies have now identified and characterized numerous TMPs involved in plant-pathogen interactions (**Table 1**) (Kumara et al., 2022). These TMPs function as Pattern Recognition Receptors (PRRs) on the plant membrane, detecting microbial molecules termed Microbe/Pathogen-Associated Molecular Patterns (MAMPs/PAMPs) — including bacterial flagellin, EF-Tu, and fungal chitin — and plant-derived molecules such as oligogalacturonides, categorized as Damage-Associated Molecular Patterns (DAMPs). This detection trigger Pattern-Triggered Immunity (PTI). The predominant classes of plant PRRs are the cell surface Leucine-Rich Repeat domain (LRR) Receptor Kinases (LRKs) and LRR Receptor Proteins (LRPs), which feature ligand-binding (e.g., LRR) and transmembrane domains (e.g., LysM). Additionally, Receptor-Like Kinase (RLKs) feature an intercellular kinase domain essential for signal transduction that leads to robust antimicrobial responses through mitogen-activated protein kinase (MAPK) cascades (Yamada et al., 2017). Pathogens can suppress the PTI by releasing effector proteins into the host cells. Additionally, in Effector-Triggered Immunity (ETI), plants counteract pathogen effector proteins through intracellular nucleotide-binding leucine-rich repeat receptors (NB-LRR), leading to defensive responses like leaf necrosis, cell death, and reactive oxygen species (ROS) release – symptoms of a hypersensitive response (HR) (Yuan et al., 2021b). TMPs enhance ETI by amplifying signal transduction and modifying ion fluxes, underscoring their vital role in plant defense mechanisms. Recent studies support the inclusion of TMPs in sustainable disease control strategies of crops, such as grapevine (*Vitis vinifera*). It is the most economically significant fruit crop cultivated worldwide that is threatened by several pathogens, such as powdery mildew (*Erysiphe necator*) and botrytis (*Botrytis cinerea*), which not only significantly reduce fruit yield and quality but also impact the global viticulture sector. It is indicative that viticulture consumes 70% of all agrochemicals used in the European Union, with associated environmental and health risks (Hollomon, 2015). Despite the challenges in identifying TMPs due to their high hydrophobicity (Tusnády et al., 2004; Nakao et al., 2020), emerging techniques are enhancing our understanding of grapevine immunity. This could have profound implications for viticulture, offering strategies to improve grapevine resilience against pathogens, supporting sustainable agriculture and crop protection efforts.

**Table 1:** List of transmembrane genes characterized in plants and involved in defense pathways.

Gene	Gene Bank ID	Defense pathway	Role in immunity	Species	Reference
Fls2	834676	PTI	Recognizes flagellin	*A. thaliana*	Gómez-Gómez and Boller, 2000
Efr	832170	PTI	Recognizes EF-Tu	*A. thaliana*	Zipfel et al., 2006
CERK1	821717	PTI	Recognizes chitin	*A. thaliana*	Miya et al., 2007
LYK4, LYK5	816909 817923	PTI	Recognizes peptidoglycan/chitin	*A. thaliana*	Willmann et al., 2011
LYK7	101258329	PTI	Recognizes peptidoglycan/chitin	*S. lycopersicum*	Zeng et al., 2012
PEPR1, PEPR2	843639 838353	PTI	Recognizes DAMPs	*A. thaliana*	Yamaguchi et al., 2006
Xa21	107276510	PTI	Recognizes Xanthomonas patterns	*O. sativa*	Song et al., 1995
RPK2	821240	PTI	Signaling	*A. thaliana*	Kinoshita et al., 2010
GHR1	827842	PTI	Calcium signaling	*A. thaliana*	Hua et al., 2012
Ve1	543659	ETI	Recognizes Verticillium effector	*S. lycopersicum*	Fradin et al., 2009
Bak1	829480	Both PTI and ETI	Co-receptor	*A. thaliana*	Li et al., 2002
BIR2	822474	Both PTI and ETI	Modulates BAK1	*A. thaliana*	Blaum et al., 2014
CRN	831170	Both PTI and ETI	Signaling	*A. thaliana*	Muller et al., 2008
CLV1, CLV2	843915 842849	Immune overlap	Recognizes PAMPs	*A. thaliana*	Clark et al., 1997
PERK1, PERK2, PERK4	822051 28719311 816362	Cell integrity	Interact with defence pathways	*A. thaliana*	Babu et al., 2008
SWEET1 SWEET11, SWEET12, SWEET13, SWEET14, SWEET10	838744 824035 832431 835152 828604 83515	Sugar transport	Plant-pathogen interactions	*A. thaliana*	Chen et al., 2010
AHA4	823950	Ion transport	Influencing stomatal closure	*A. thaliana*	Vitart et al., 2001
RBOHA, RBOHB RBOHD RBOHE RBOHF RBOHG RBOHH RBOHI RBOHJ	842710 837430 835179 838506 842710 828612 836123 826725 823724	ROS production in immune responses	Recognizes *Phytophthora infestans* and *P. syringae.*	*A. thaliana*	Torres et al., 2002; Sagi and Fluhr, 2006; Cheng et al., 2013
SDIR1	100246103	ABA pathway	Enhances the production of ABA	*V. vinifera*	Tak and Mhatre, 2013

PTI, pathogen triggered immunity; ETI, effector triggered immunity.

This review synthesizes the current research on TMPs identified in *V. vinfera* and knowledge on their role in response to the most significant threats to grapevine. We first provide an overview of these genes and then examine various methodologies used to investigate TMP structure, function and regulation, highlighting their advantages and limitations. Our aim is to identify research gaps and suggest future research directions, providing a robust framework to enhance our understanding of crop immunity and promote sustainable agricultural practices.

## 2 TMP-encoding genes involved in grape biotic stress response

**Figure 1:**
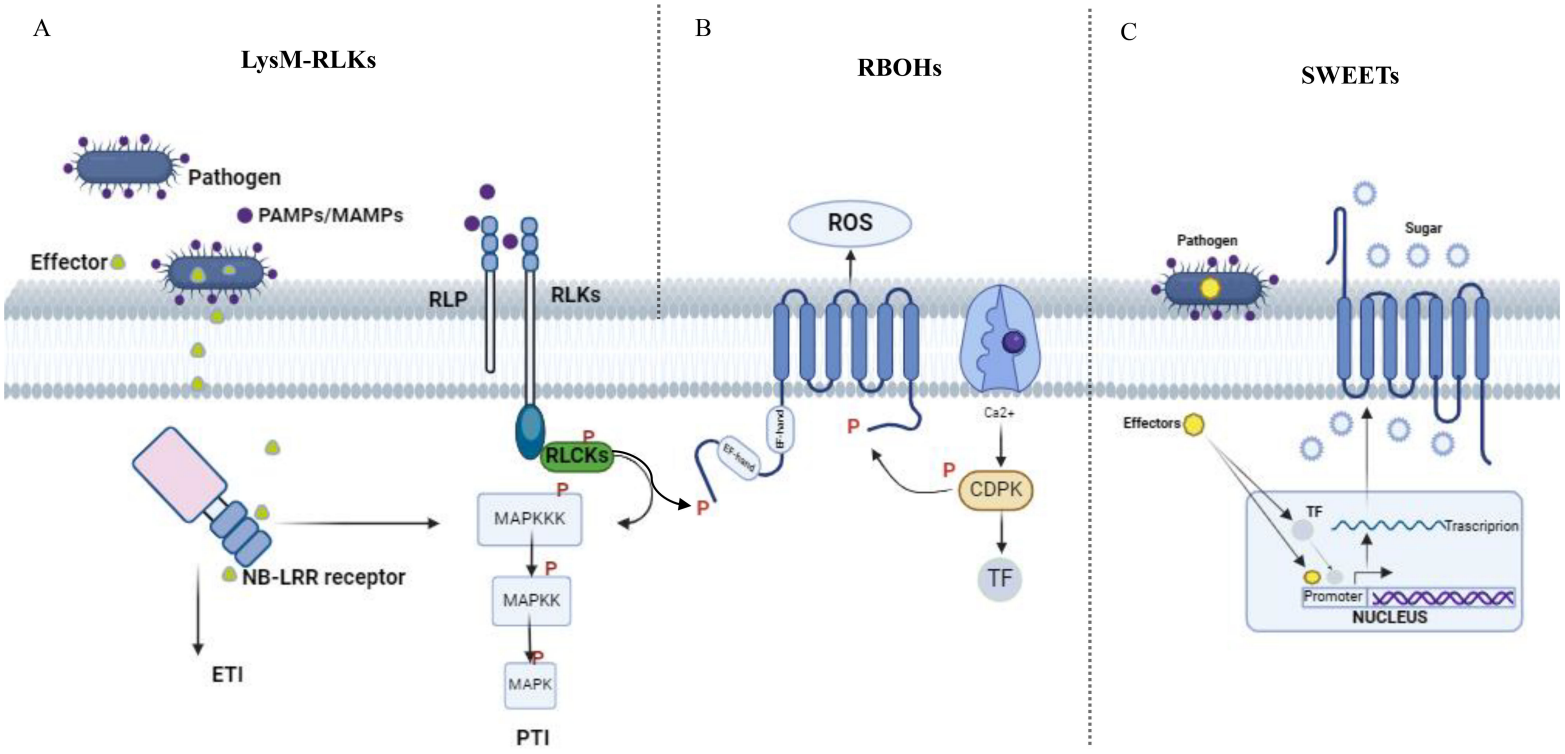
Overview of signalling mediated by plant after pathogen infection. After a plant is infected by a pathogen, triggers a signaling process. This begins with the recognition of Pathogen/Microbe-Associated Molecular Patterns (PAMPs/MAMPs) by pattern recognition receptors (PRRs) **(A)**, which interact dynamically with co-receptors and receptor-like cytoplasmic kinases (RLCKS). Transphosphorylation occurs within the PRR complexes, initiating downstream signaling. These signals, originating from PRRs, are transmitted through phosphorylation cascades involving mitogen-activated protein kinases (MAPKs) and calcium-dependent protein kinases (CDPKs). This signaling pathway ultimately affects downstream targets like the NADPH oxidase RBOHD **(B)** during Pattern-Triggered Immunity (PTI). Additionally, many pathogens rely on glucose from host plants as a carbon source for their growth before they can successfully invade. Upon invading plants, pathogens release TAL effectors into host plant cells. These effectors prompt the expression of plant SWEETS **(C)**, either directly or indirectly through the activation of transcription factors. As a result, sugar flows into the apoplast, providing nutrition for the pathogens.

To date, our understanding of TMPs involved in biotic stress in grapevines remains limited. Notably, only three of them have been studied for their involvement in pathogen infection reaction, namely the Lysin-Motif Receptor-Like Kinase (LysM-RLK)(**Table 2**), the plant respiratory burst oxidase homolog (Rboh) (**Table 3**), and the Sugar Will Eventually Be Exported Transporter (SWEET) (**Table 4**; **Figure 1**) (Baker et al., 2012; Cheng et al., 2013; Brulé et al., 2019). The following paragraph will delve into the current knowledge on each of these gene families, examining their specific roles in plant defence mechanisms and their potential applications in enhancing grapevine resistance to biotic stresses.

**Table 2:** Number of LysM-RLK family members characterized in plant species.

Plant Species	Number of LysM-RLKs Genes	References
*A. thaliana*	5	Zhang H. et al., 2009; Arrighi et al., 2006
*Oryza sativa*	11	Hayafune et al., 2014
*Medicago truncatula*	6	Arrighi et al., 2006
*Lotus japonicus*	20	Ruman and Kawaharada, 2023
*Glycine max*	13	Yao et al., 2023
*Vitis vinifera*	6	Roudaire et al., 2023
*Brassica rapa*	8	Abedi et al., 2021
*Citrus sinensis*	9	Li et al., 2021
*Solanum tuberosum*	10	Nazarian-Firouzabadi et al., 2019
*B. juncea*	4	Yang et al., 2020
*Brassica napus*	16	Abedi et al., 2021
*Malus domestica*	4	Zuo et al., 2017

**Table 3:** Number of Rboh family members characterized in plant species.

Plant Species	Number of Rboh Genes	References
*A. thaliana*	10	Zimmermann et al., 2004
*Oryza sativa*	9	Kaur and Pati, 2016
*Glycine max*	17	Liu et al., 2019
*Zea mays*	9	Zhang et al., 2023
*Solanum melongena L*.	8	Du et al., 2023
*Vitis vinifera*	8	Cheng et al., 2013
*Medicago truncatula*	14	Marino et al., 2011
*Brassica rapa*	14	Li et al., 2019
*Capsicum annuum*	8	Zhang et al., 2021
*Tricutum aestivum*	17	Wang et al., 2018

**Table 4:** Number of SWEET family members characterized in plant species.

Plant Species	Number of SWEET Genes	References
*A. thaliana*	17	Chong et al., 2014
*Oryza sativa*	21	Yuan and Wang, 2013
*Vitis vinifera*	19	Chong et al., 2014
*Zea mays*	24	Zhu et al., 2022
*Glycine max*	52	Patil et al., 2015
*Solanum lycopersicum*	29	Filyushin et al., 2023
*Brassica rapa*	27	Wang et al., 2018
*Citrus sinensis*	18	Yin et al., 2023
*Medicago truncatula*	25	Hu et al., 2019
*Solanum tuberosum*	35	Manck-Götzenberger and Requena, 2016
*Triticum aestivum L.*	59	Qin et al., 2020

### 2.1 Lysin motif receptor like kinases

**Figure 2:**
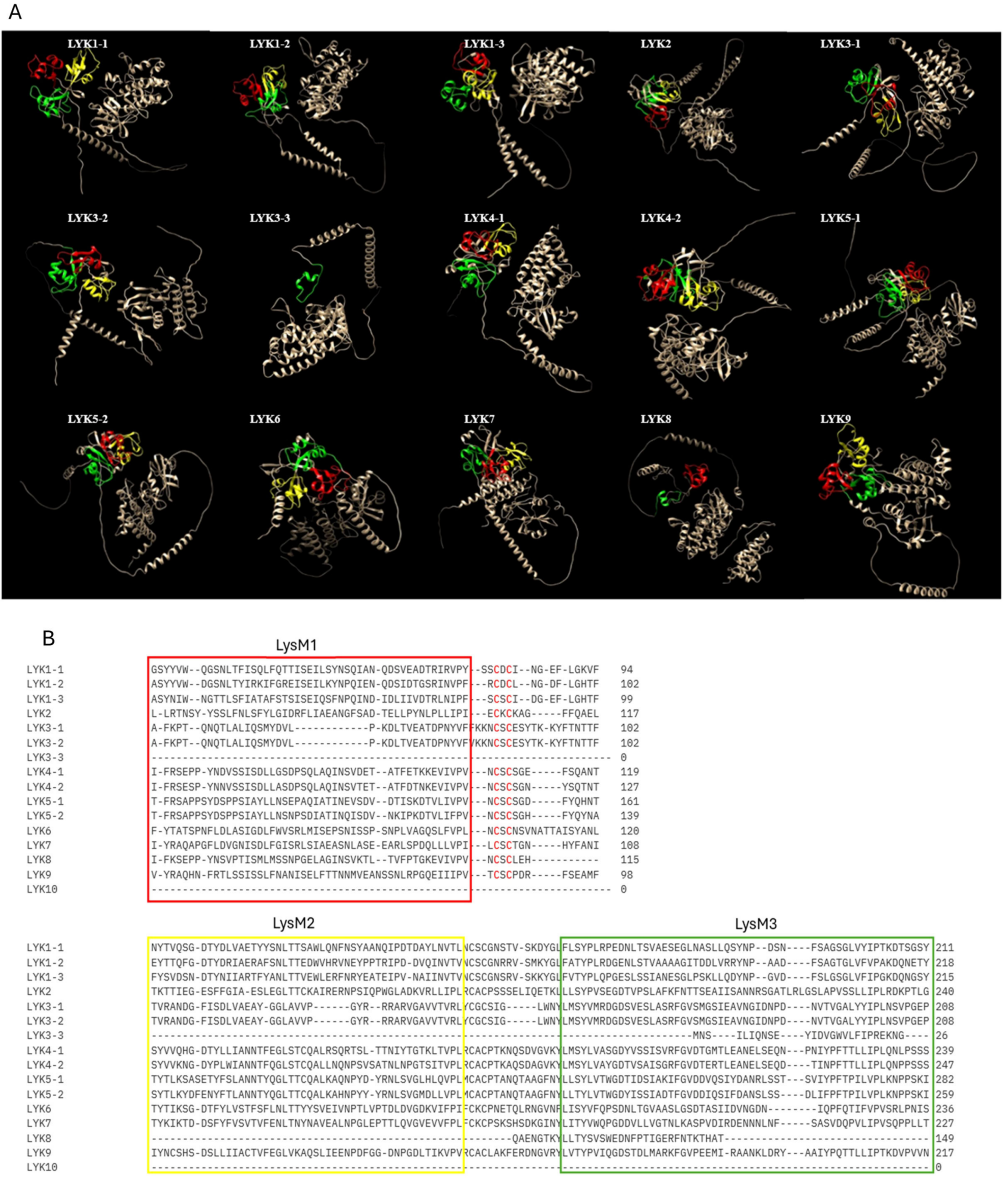
**(A)** AlphaFold prediction of the structure of the LysM-RLKS (LYK) proteins performed with ChimeraX AlphaFold, each LysM domain is represented in different colour: LysM1 (red), LysM2 (yellow) and LysM3 (green). **(B)** Sequence alignment of conserved LysM domains located within N-terminal domain of LYKs protein.

Lysin Motif Receptor Like Kinases (LysM-RLKs) represent one of the major classes of PRRs (Gust et al., 2012). Upon binding to PAMPs/MAMPs, LysM-RLKs initiate signalling cascades that lead to the activation of defense responses in plants. These responses may include the production of ROS, activation of defense genes, and reinforcement of the PTI (Buendia et al., 2018) (**Figure 1**). Beyond their function in immunity, they participate in symbiotic interactions, specifically recognizing signals from beneficial microbes like mycorrhizal fungi and rhizobia. Through this recognition, LysM-RLKs facilitate the formation of symbiotic partnerships, resulting in increased nutrient absorption, heightened stress resilience and overall enhancement of plant growth and development (Chiu and Paszkowski, 2020; Cope et al., 2023). Their proteins contain an extracellular ligand-binding domain, a transmembrane domain, and an intracellular kinase domain (**Figure 2**). The extracellular domain includes one to three LysM domains, each approximately forty amino acids long, with a conserved βααβ structure, crucial in detecting and responding to external stimuli (Bellande et al., 2017, Zhang X.C. et al., 2009). Between the LysMs of all plants are found highly conserved cysteine pairs separated by one amino acid (CXC) that participates to the structural stability of the domain (**Figure 2**). The transmembrane region ensures the protein correct positioning within the cell membrane, supporting its overall functionality (Tombuloglu et al., 2019). Lastly, the intracellular kinase domain typically encompasses conserved motifs or residues necessary for ATP hydrolysis (Tanaka et al., 2013) and catalyzes reactions crucial for downstream signalling (Zeng et al., 2012). LysM-RLKs were first discovered in *A. thaliana* (Shiu and Bleecker, 2001) and subsequently in other plant species, including *V. vinifera* (**Table 2**). LysM-RLKs can be divided into two major classes, the LYKs (Lysin Motif Receptor Kinase) in which the kinase domain is active and the LYRs (LysM-containing Receptor-like Protein) with an inactive pseudokinase domain (Schwessinger et al., 2011). In 2007, Miya et al. discovered CERK1, an RLK that is essential in the recognition and transmission signals from the chitin oligosaccharide elicitor in *A. thaliana*. Subsequent studies have highlighted LYK4 and LYK5 as pivotal for chitin signalling in this species (Wan et al., 2008; Shimizu et al., 2010; Cao et al., 2014; Xue et al., 2019). In particularly, AtLYK5 binds strongly to chitin and is proposed to form dimers with AtLYK4 upon ligand binding (Cao et al., 2014). However, neither receptors can transduce signals independently; AtLYK5 must interact with AtCERK1/LYK1, which carries a functional kinase domain (Xue et al., 2019). A comparable molecular complex model involving the LysM-RLK OsCERK1 and the LysM proteins OsCEBIP, OsLYP4, and OsLYP6 has been suggested for chitin perception in rice (Shimizu et al., 2010). Recently, research on grapevine has confirmed the involvement of LysM-RLKs in immune response activation (Brulé et al., 2019; Roudaire et al., 2023). The latest annotation of the grapevine genome indicates the presence of 16 VvLYK genes, which encode 16 LysM-RLKs proteins (Roudaire et al., 2023). Among the orthologs of AtCERK1/LYK1, only VvLYK1-1 and VvLYK1-2 have been demonstrated to facilitate the perception of PAMPs/MAMPs in grapevine. As in *A. thaliana* and rice, VvLYK1-1 and VvLYK5-1 interact only after chitin recognition (Brulé et al., 2019). Roudaire et al. (2023) observed an up-regulation of VvLYK5-1 in grapevine leaves after *E. necator* attack, indicating its involvement in chitin perception. Interestingly, both VvLYK5-1 and VvLYK5-2 lack most of the amino acids necessary for kinase activity, like AtLYK5, implying that the functional kinase domain is absent in VvLYK5-1 and VvLYK5-2. Consequently, it is proposed that VvLYK5 may interact with a co-receptor to transmit signals, thereby activating chitin-induced immunity (Roudaire et al., 2023). Chitin is a well-known PAMP recognized by LYK proteins in plant cells infected by fungi. However, chitin perception mechanisms in grapevine remain unexplored, particularly regarding the genetic diversity of LysM-RLK genes among various grapevine cultivars. Xue et al. (2019) demonstrated that *A. thaliana* perceives chitin via a receptor complex composed of LYK1, LYK4 and LYK5. It would be interesting to investigate if a similar tripartite chitin receptor complex involving VvLYK1-1, VvLYK4-2 and VvLYK5-2 exists in *V. vinifera*. Resolving the entire mechanism of chitin and chitosan perception in grapevine holds significant agricultural importance, as this knowledge, could enhance disease resistance breeding program, promoting sustainable grape production practices.

### 2.2 Plant respiratory burst oxidase homolog genes

**Figure 3:**
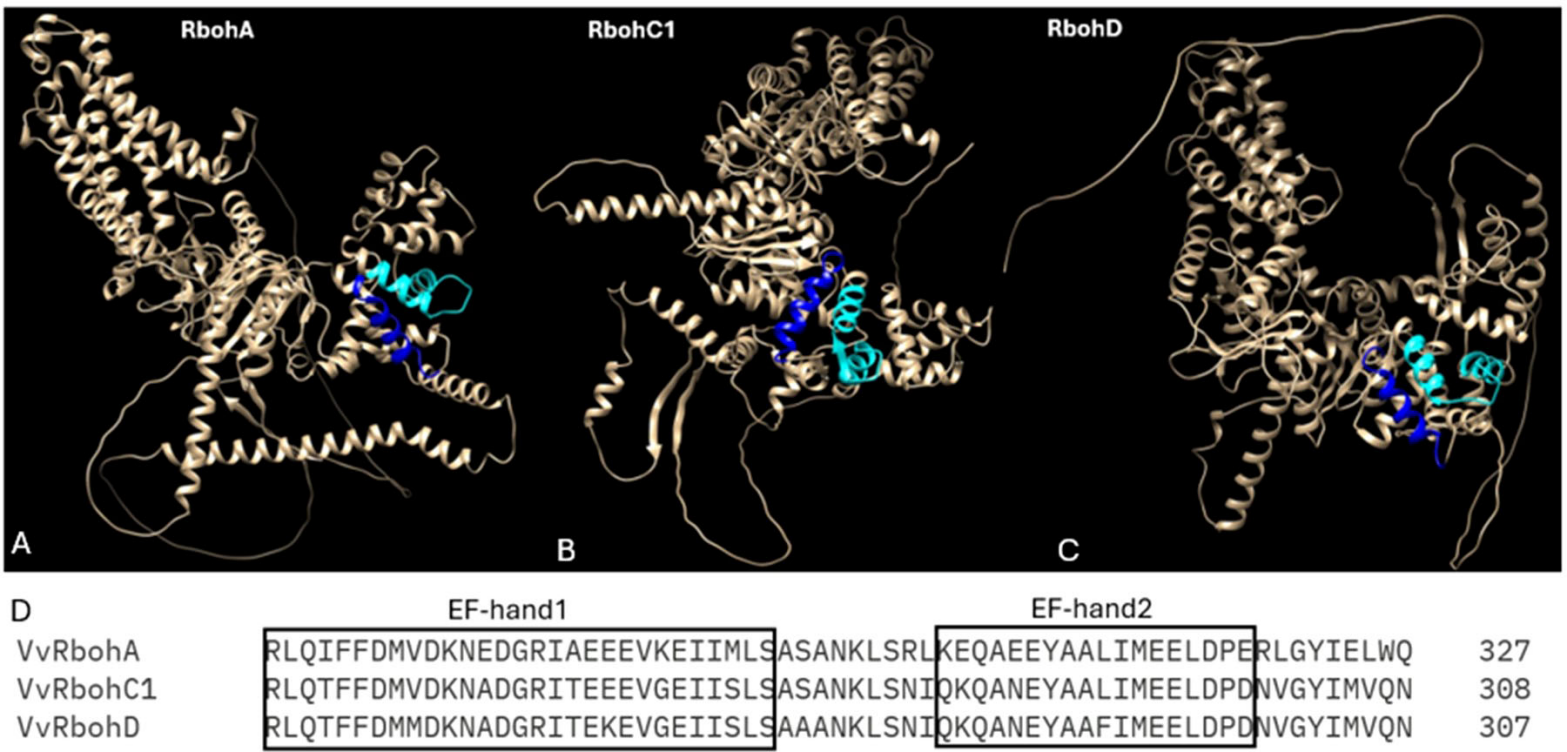
AlphaFold prediction of the structure of the RBOHs proteins performed with ChimeraX AlphaFold, each EF-hand domain is represented in different colour: EF-domain1 (cyan) and EF-hand domain2 (blue). **(A)** The 3D model of VvRbohA **(B)** the 3D model of VvRbohC1 **(C)** the 3D model of VvRbohD **(D)** Sequence alignment of conserved two EF-hand domains located within N-terminal extension.

Plant cells respond to biotic stress by accumulating reactive oxygen species (ROS), a critical component of PTI and ETI. One key ROS, hydrogen peroxide (H_2_O_2_), primarily forms through the conversion of superoxyde, a reaction catalysed by the enzyme respiratory burst oxidase homolog (Rboh) protein in the apoplast (Liu et al., 2016; Navathe et al., 2019). Rboh proteins are integral membrane proteins characterized by six conserved transmembrane helices. They feature two EF-hand calcium-binding domains in their N-terminal region, regulated directly by Ca²^+^ ions. The C-terminal region consists of a hydrophilic domain with binding sites for flavin adenine dinucleotide (FAD) and NADPH, oriented towards the cytosol. In the apoplast, heme groups facilitate electron transport across the membrane to O_2_, the electron acceptor, through FAD (Mahalingam et al., 2021) (Chapman et al., 2019) (**Figure 1**). When plants encounter pathogens, they produce ROS via Rboh activity as part of their defense mechanisms (Mittler, 2017; Mansoor et al., 2022; Ali et al., 2023). The Rboh gene family, which is conserved across both angiosperms and gymnosperms, include several subgroups that have been well-characterized (Zhang et al., 2023). The rice rbohA gene, the first Rboh gene identified in plant, shares homology with the mammalian gene gp91^phox^ (Groom et al., 1996). Since this discovery, Rbohs genes have been identified across various plant species, with a variable number of family members (**Table 3**). In grapevine, 7 Rboh proteins have been identified, with VvRbohA, VvRbohC1 and VvRbohD localized in the plasma membrane and the others predicted to be in the chloroplast thylakoid membrane. These proteins have N-terminal sequences containing two potential Ca^2+^-binding EF-hand motifs, critical for their regulation (**Figure 3**) (Cheng et al., 2013). Notably, VvRbohD shows significant up-regulation after powdery mildew inoculation, highlighting its potential role in biotic stress responses (Cheng et al., 2013; Rivas et al., 2023). In other species, such as eggplant, it has been shown that SmRbohB significantly increases ROS production upon *Verticillium dahliae* treatment, restricting pathogen growth and highlighting its potential as a gene for stress tolerance (Du et al., 2023). These results mark early steps in studying Rboh gene functions in grape, pointing to the need for further research to fully understand their contributions to stress resistance.

### 2.3 Sugars will eventually be exported transporters genes

**Figure 4:**
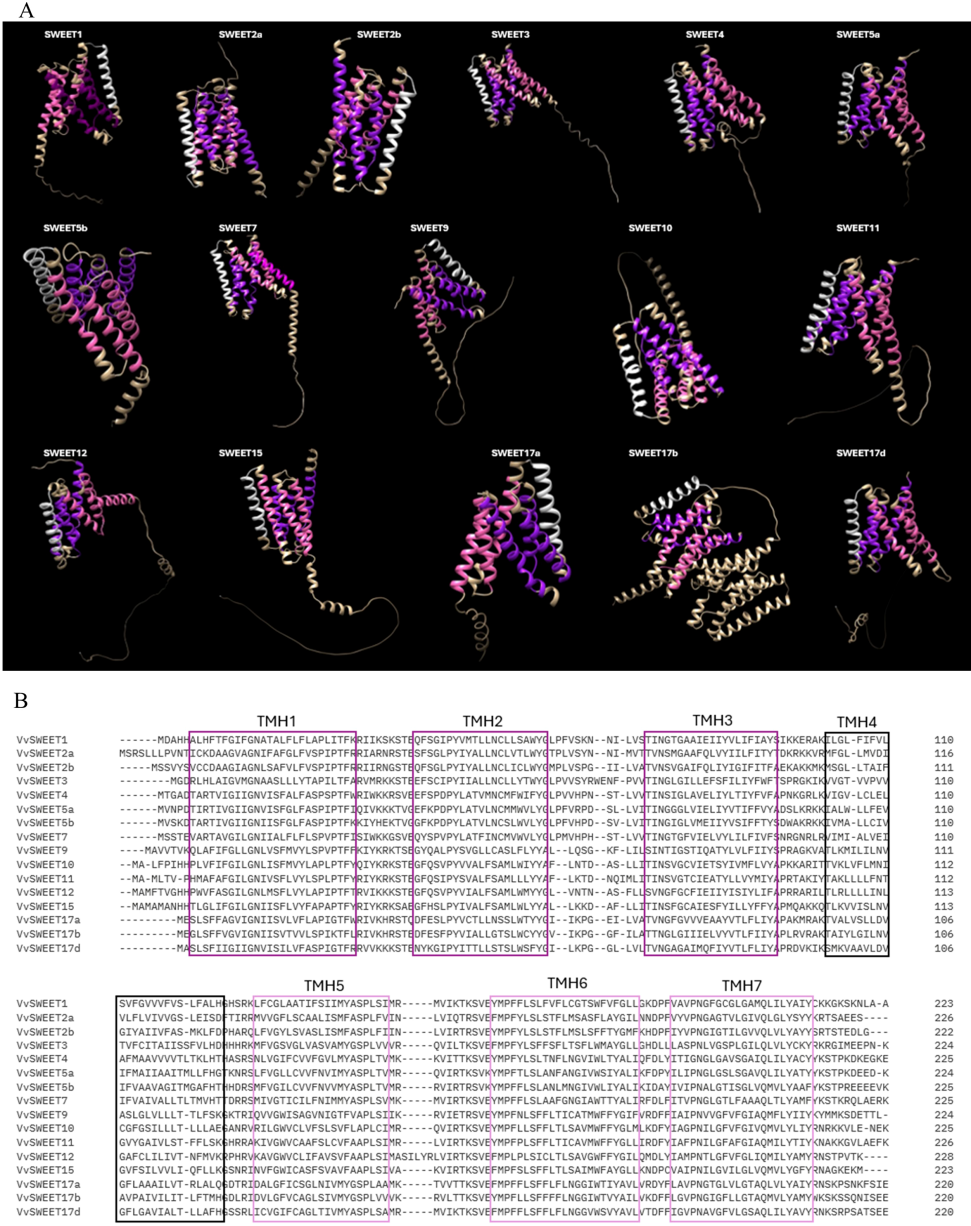
**(A)** AlphaFold prediction of the structure of the SWEETS proteins performed with ChimeraX AlphaFold, Each domain which comprises three transmembrane helices (3-TMs) is represented in different colour: The first domain is purple, the second domain is pink and the fourth transmembrane helix (TMH), which is less conserved and divides the SWEET protein into two domains is white. **(B)** Sequence alignment of the seven transmembrane domains of SWEET proteins.

The Sugar Will Eventually be Exported Transporter (SWEET) genes engage in sugar transport and distribution in plants, playing a significant role in plant physiology (Eom et al., 2015). These genes are part of the MtN3/saliva family and consist of seven transmembrane α-helices (TM1 to TM7). An ancient duplication resulted in their unique 3-1-3 configuration, forming a central channel for sugar transport. Both the N-terminal and C-terminal ends of the SWEET protein are cytoplasmatic (Chen et al., 2010) (**Figure 4**). SWEET genes are highly conserved across different plant species and categorized into four clades, each specializing in the transport of specific sugars: hexoses (Clades I and II), sucrose (Clade III), and fructose (Clade IV). These proteins are expressed in various tissues – leaves, seeds, roots, and flowers – and regulate important physiological processes such as pollen development, fruit ripening, and leaf senescence (Chen et al., 2012; Gautam et al., 2022; Ko et al., 2022). Their expression can be induced by biotic stresses (Zhu and Gong, 2014; Zhu et al., 2019). In various plant species, including *V. vinifera*, SWEET homologs have been identified as susceptibility genes, acting as targets of effector proteins during host-microbe interactions. Indeed, some pathogens hijack SWEET genes to manipulate sugar flow and create nutrient-rich environments for their invasion and growth (Baker et al., 2012). On the other hand, some SWEETs can also function as resistance genes, promoting resistance to certain pathogens (Kang et al., 2023). AtSWEET1 from *A. thaliana* was the first SWEET protein to be identified in plants (Chong et al., 2014). Later, SWEETs were identified in many other plant species (**Table 4**) such as *M. truncatula* (Hu et al., 2019), *O. sativa* (Yuan and Wang, 2013)*, S. tuberosum* (Manck-Götzenberger and Requena, 2016), *T. aestivum* (Qin et al., 2020) and *G. max* (Patil et al., 2015). Research has shown that different SWEET genes in plants are upregulated in response to powdery mildew infection, indicating their crucial role in biotic stress resistance. Chong et al. (2014) characterized the SWEET family in grape genome with VvSWEET4 particularly upregulated after *B. cinerea* infection. More recently, Zhong et al. (2024) identified notable differences in VvSWEET gene expression between resistant (Shangyou, Beihong, Beimei and Gold Finger) and susceptible (Red Globe, Gebixinxiu, Thompson Seedless and JingXiangyu) grape varieties. VvSWEET1 and VvSWEET10b exhibited elevated expressions in the latter, while VvSWEET3 was more expressed in the former, suggesting a potential role for these genes in the response to *B. cinerea* infection. Chen et al. (2010) found an induction in AtSWEET12 during powdery mildew infection in *A. thaliana*, while Pan et al. (2024) observed significant upregulation of AsSWEET1a, 3a, 11, and 16 in *Avena sativa*. Additionally, Chong et al. (2014) highlighted thew role of AtSWEET4 in resistance to *B. cinerea*, demonstrating that atsweet4 knockout mutants exhibited resistance. Given the significant role of soluble sugar content in determining the yield and economic value of grape berries, the efficient sugar partitioning is crucial in grapevine. These findings underline the potential of SWEET genes in crop improvement strategies aimed at enhancing yield and stress resistance through biotechnological manipulation of sugar transport mechanisms.

## 3 Methods used to examine transmembrane proteins

Understanding the structure and function of TMPs is crucial for unraveling their roles in various biological processes and guiding targeted breeding programs. However, studying these integral membrane proteins presents unique challenges, such as low expression levels and the difficulties associated with detergent-based structural studies (Thoma et al., 2018). To overcome these hurdles, researchers have developed a suite of assays that, when used in combination, can provide a comprehensive understanding of TMP structure, function, and regulation across various physiological contexts (Boulos et al., 2023). In this section, we delve into *in vitro*, *in vivo* and *in silico* techniques that can be employed to study TMPs in plants offering researchers a valuable resource for selecting the most appropriate strategies in TMP research. A summary of the techniques considered with strengths and limitations is reported in **Table 5**.

**Table 5:** *In vitro*, *in vivo* and *in silico* techniques mainly used for protein structure and interaction studies.

Techniques	Advantages	Limitations
*In vitro*		
X-ray diffractometry	Gold standard in protein structure Structural information High resolution image Widespread applicability	Need high-quality crystals Potential radiation damage Failed for complex protein Instrumentation and cost
NMR spectroscopy	Structural information Non-destructive and non-invasive No requirement for crystallization	Sensitivity Size limitations Instrumentation and cost Sample conditions
Electron cryo microscopy	Structural information No requirement for crystallization Native state preservation Large protein application	High cost and expertise Potential radiation damage Failed for small protein
Isothermal Titration Calorimetry	Gold standard in protein interaction Interaction information Label-free technique Quantitative and direct measurement No binding models assumptions Suitable for small and large molecules Protein and DNA interactions	Lower sensitivity for weak interactions Selection of appropriate experimental conditions Only for 1:1 interaction
Surface plasmon resonance	Interaction information Real-Time monitoring Label-Free technique Quantitative analysis High sensitivity	Sample complexity Mass sensitivity Information about conformational changes
Fluorescence polarization	Interaction information Sensitivity Real-Time measurements Quantitative information	Failed for big protein Selection of appropriate experimental conditions Instrumentation
*In vivo*		
Fluorescence resonance energy transfer (FRET)	Interaction information Real-Time monitoring High sensitivity Quantitative information Non-invasive	Spectral overlap Intracellular mobility Selection of appropriate experimental conditions
Yeast two hybrid (Y2H)	Interaction information High-throughput screening Genome-wide studies trough libraries	Selection of appropriate experimental conditions False positives and negatives Can fail for TMP
Mating-based split biquitin system (mbSUS)	High sensitivity High specificity Versatility Suggested for TMP	Selection of appropriate experimental conditions False positives
Bimolecular fluorescence complementation (BiFC)	Interaction information Cellular localization information Non-invasive Direct visualization	Artifacts Influence of fusion proteins
*In silico*		
Topology prediction	Localization information Orientation information Predictions of Alpha-helical topology Predictions of Beta-barrel topology	Overall accuracy max ∼80% Erroneous prediction of signal peptides in TMPs Non-standard topological features
AI-based prediction methods and deep learning	3D structure from aa sequence High confidence prediction of loop regions and intracellular N- and C-terminal regions	low confidence for very flexible regions low confidence for TM connecting segments
Protein function prediction	Interaction formation Discernment between native and unreliable complex models Membrane protein systems building simulation	Availability of 3D protein structure No flexibility allowed for model partners

Their advantages and limitations are also reported.

### 3.1 *In vitro* techniques

*In vitro* techniques are experimental methods conducted outside of a living organism, typically in a controlled laboratory setting, used to simplify complex systems and make it easier to identify and analyze specific structures or interactions. The main *in vitro* techniques for TMP studies include the X-ray diffractometry, the Nuclear Magnetic Resonance (NMR), and the electron cryo-microscopy (Cryo-EM). Additionally, other *in vitro* techniques can be used to study protein functions and interactions, such as the isothermal titration calorimetry, the surface plasmon resonance and the fluorescence polarization. Hereunder, we provide a critical analysis of their principles, applications, strengths, and limitations for TMPs investigation.

#### 3.1.1 X-ray diffractometry

X-ray diffractometry is a technique used to determine the 3D structure of proteins by diffracting X-rays through protein crystals. The diffraction of the X-rays provides exceptional resolution (<1.5 Å), enabling precise determination of atomic positions. While this technique is widely employed in grape protein research, it has not been applied to the study of grape TMPs yet. One of the primary limitations of X-ray diffractometry is the difficulty in obtaining highly purified proteins in crystalline form, especially for TMPs. The main challenge is that purifying and crystallizing TMPs is difficult because they do not fold correctly in normal aqueous (water-based) environments, which traditional crystallization methods depend on. Special approaches are required to work with TMPs since they behave differently from water-soluble proteins. A successful strategy for determining the structure of TMPs is the use of detergents (Tate, 2010). This approach has been effective in determining the crystallographic structure of a CMP-sialic acid transporter from *Zea mays* (Nji et al., 2019) and a multidrug efflux transporter from *Camelina sativa* (Tanaka et al., 2017).

#### 3.1.2 Nuclear magnetic resonance

NMR can determine the 3D structure of proteins in solution, revealing the arrangement of atoms and chemical bonds. Proteins are typically dissolved in a suitable solvent and placed in a strong magnetic field, causing the nuclei of atoms to align. Radio frequency (RF) pulses are then applied to the sample, perturbing the alignment of the nuclei. As the nuclei relax back to their equilibrium state, they emit detectable NMR signals, which are processed and analyzed to reconstruct the protein structure. While NMR is widely used for studying proteins, its application to transmembrane proteins (TMPs) is limited (Yeh et al., 2020), similar to X-ray diffractometry. This limitation arises from the size and structural characteristics of TMPs, as NMR typically requires smaller proteins and a significant amount of sample due to its sensitivity constraints (Xu et al., 2007). These factors make studying large TMPs challenging. However, NMR has the advantage of being able to simulate the membrane environment in solution, making it potentially valuable for TMP research. Despite these advantages, no NMR studies have been published specifically on grape TMPs. However, a few studies have focused on portions of TMPs in plants. For instance, a fully functional C-terminally truncated twin-arginine translocase (Tat) from *A. thaliana*, consisting of 53 amino acids, was investigated using solution-state NMR in micelles and lipid bilayers (Pettersson et al., 2018). Another example is the chloroplast outer envelope channel OEP21 from *Pisum sativum*, a protein of 187 amino acids, whose structure was solved using solution NMR, revealing a beta-barrel pore structure (Günsel et al., 2023).

#### 3.1.3 Electron cryo-microscopy

In the past 10 years, cryo-EM has emerged as a revolutionary technique in structural biology, enabling researchers to visualize biological molecules, including TMPs, at near-atomic resolution (Wang, 2022; Castells-Graells et al., 2023). This technique involve the use of biological samples rapidly frozen in a thin layer of vitreous ice to preserve their native state and the frozen sample is then imaged using an electron microscope. Electrons interact with the sample, creating multiple 2D projection images that are collected from different orientations of the sample to reconstruct the 3D structure of the molecule. In 2019, the number of membrane proteins structurally resolved by cryo-EM surpassed those resolved by X-ray diffractometry (García-Nafría and Tate, 2021), indicating that cryo-EM has become the most effective technique for resolving the 3D structures of even large proteins, typically those exceeding 50 kDa in size. In relation to plant TMPs, over 200 structures have been deposited in databases in the past five years. The majority of these involve channel structures, such as potassium, calcium, chloride, and glutamate receptor-like channels (Dickinson et al., 2021), as well as transporters, including ABC and sodium transporters (Huang et al., 2024; Ying et al., 2024).

#### 3.1.4 Isothermal titration calorimetry

Isothermal titration calorimetry (ITC) is widely used for characterizing the binding of small compounds to larger macromolecules as DNA or proteins. It has become the gold standard for studying molecular interactions in solution measuring the heat released or absorbed during protein-ligand interactions by a microcalorimeter. This instrument has two cells that need to be maintained at the same temperature: one containing only buffer and the other containing the sample. By gradually adding small amounts of ligand, any interaction with the target sample will cause a detectable heat variation. This method allows researchers to define a complete thermodynamic profile of the interaction, providing data on enthalpy and entropy changes, binding constants, and reaction stoichiometry. Overall, ITC has the advantage of providing direct and quantitative measurements and is highly versatile in the types of reactions it can study. However, it has lower sensitivity for weak interactions and is most applicable to 1:1 interactions (Falconer, et al., 2021). ITC has already been used to study the interactions of medical compounds with three major classes of membrane proteins: G-protein coupled receptors, ion channels, and transporters (Draczkowski et al., 2014). In plants, it has been employed for several purposes, for example to test the binding capability of the Ferredoxin-NADP+ reductase to the cytochrome b6f complex from *Spinacia oleracea* and *Z. mays* in the electron transport chain of oxygenic photosynthesis (Cramer, 2022) or to test the interaction between the alkaline isoform of the PR-5 protein, purified from soybean hulls, and the CM-pachyman or bilayer vesicle to explain its antimicrobial activity (Liu et al., 2016).

#### 3.1.5 Surface plasmon resonance

Surface plasmon resonance (SPR) spectroscopy enables the real-time characterization of binding affinity and kinetics for membrane protein-ligand interactions, using relatively small amounts of membrane protein in a native or native-like environment. The SPR technique typically requires the target molecule to be immobilized on a sensor chip while the analyte in solution flows over the sensor surface. The binding of biomolecules results in a signal that depends on changes in the refractive index at the sensor surface (Patching, 2014). The advantages of SPR include real-time kinetic measurements, high sensitivity, and the ability to provide binding affinities and association/dissociation rates for label-free molecules. However, limitations include weak signals when analyzing small molecules, the need for high-purity analytes, and complications from protein conformational changes (Dobrovodský and Di Primo, 2023). Additionally, non-specific signals may require inhibitors or competitive molecules, complicating data analysis, and careful experimental control is needed to prevent unwanted analyte binding to the sensor surface. SPR is widely applied to study a broad range of membrane proteins and can also be used for various biomolecular interactions, including protein-DNA, protein-protein, protein-carbohydrate, protein-RNA, and protein-lipid interactions. In plants, SPR has been employed to investigate G-protein-coupled receptor (GPCR) transmembrane proteins and molecular interactions involved in various aspects of plant development, such as protein-carbohydrate interactions, protein-chaperone interactions, phytohormone signaling detection, and plant-virus diagnostics (Jain et al., 2016).

#### 3.1.6 Fluorescence polarization

Fluorescence polarization (FP) is a non-disruptive technique that allows rapid, quantitative analysis of enzyme activities and molecular interactions by measuring the binding of a fluorescently labeled ligand to a larger molecule. A freely rotating small molecule with a fluorescent probe emits depolarized light. When it binds to a larger target, its rotation slows, causing the emitted light to become more polarized. FP detects these changes in light polarization (Hall et al., 2016). In its variant, single-molecule fluorescence polarization (SMFP), the technique also reveals the structural basis of protein activity and tracks conformational changes between distinct states (Forkey et al., 2000). The ability to study molecular processes in solution, along with time-course readability and a homogeneous assay format, makes FP particularly suitable for high-throughput screening. However, FP has limitations. It is highly dependent on the size difference between the interacting molecules and on the choice of fluorescent label. If the size difference is too small, the method may not perform reliably. Additionally, the fluorescent probe can interfere with ligand binding or create nonspecific interactions. Low-affinity interactions can also skew results, as high concentrations of unlabelled protein can lead to artificial crowding effects (Dharadhar et al., 2019). FP has been used to study transmembrane GPCRs, avoiding the need for radioactive methods previously required to monitor binding events (Burke et al., 2003). High-throughput FP assays have also been developed to quantify biotin and biotin-binding proteins in *Nicotiana tabacum* leaf extracts (Martin et al., 2008) and to screen ligands of the jasmonate COI1–JAZ co-receptor in Arabidopsis thaliana (Takaoka et al., 2019).

### 3.2 *In vivo* techniques

Unlike *in vitro* methods, *in vivo* approaches enable for observation of proteins in their natural state within living cells, revealing their dynamic interactions and functional roles. The techniques we have explored in depth include Fluorescence Resonance Energy Transfer (FRET), the Yeast Two-Hybrid (Y2H) system, and Bimolecular Fluorescence Complementation (BiFC). These methods are emphasized due to their accessibility and their effective combination for achieving optimal results, marking considerable progress in structural biology.

#### 3.2.1 Fluorescence resonance energy transfer

FRET is a powerful technique used to study interactions proteins (Förster, 1948). It relies on the non-radiative energy transfer between a donor and an acceptor fluorophore, which occurs when they are in proximity (typically within 1-10 nm). When the donor fluorophore, absorbing light at a specific wavelength, is excited, it can transfer energy to the acceptor fluorophores if they are within the critical FRET distance; this leads to fluorescence emission from the acceptor while the donor fluorescence intensity decreases (Periasamy, 2001). FRET is a versatile technique widely used across various biological systems such as plant (Bücherl et al., 2014), mammalian (Mitchell et al., 2017), yeast (Skruzny et al., 2019) and bacteria (Majoul, 2004). Among numerous studies, Fliegmann et al. (2016) employed FRET to reveal the formation of a complex between MtLYR3 and MtLYK3 at the plasma membrane in *N. benthamiana* leaf cells. Similarly, Roudaire et al. (2023) used FRET to demonstrate a chitin-dependent interaction between VvLYK5-1 and VvLYK1-1. Despite FRET efficiency decreasing rapidly as the distance between the donor and acceptor increases, the popularity of FRET is due to its advantages. These include high sensitivity, real-time analysis capabilities, and its non-invasive nature, eliminating the need for disrupting cellular processes.

#### 3.2.2 Yeast two hybrid

Y2H system is a method used for the analysis of protein-protein interactions in yeast. This assay is based on the ability of transcription factors to positively regulate a responsive gene for the growth on selective medium and to be organized into two separable functional domains such as the DNA-binding domain (BD) and the activation domain (AD) (Bai and Elledge, 1997). These domains can be separated and combined in a reversible way. If physically separated, the BD and AD cannot transcript the responsive gene (Causier, 2004); when recombined, they reconstitute an active transcription factor (Paiano et al., 2018). In the Y2H, given two proteins of interest, named “bait” and “prey”, two vectors are produced fusing the BD to the bait and the AD to the prey. After expression of the two proteins in yeast cells, if the pray and bait interact, BD and AD will combine reconstituting an active transcription factor and the yeast will grow on the selective media (Paiano et al., 2018). The Y2H technique is a versatile and widely used method for studying protein-protein interactions in plant (Shimizu et al., 2010) and human (Smirnova et al., 2022). However, it has limitations, particularly with membrane proteins due to their localization. Furthermore, Y2H may not be suitable for analyzing interactions that require specific cofactors or post-translational modifications not present in the yeast system. Additionally, it can be sensitive to false positives or negatives. A strategy to overcome the challenges associated with studying TMPs is to use their N-terminal and C-terminal portions as reported by Smirnova et al. (2022) in a study about human protein interaction. Another adaptation of Y2H for the study of TMPs is the split-ubiquitin system. In this method, ubiquitin is split into two halves and the bait and prey are fused to them. Their interaction reassembles ubiquitin, triggering the release of a transcription factor that then activates the expression of a reporter gene (Thaminy et al., 2004). Through this method, Wang et al. (2020) highlighted the interaction of VviABCG14 with VviABCG7, suggesting a role in cytokinin transport, important hormones involved in many aspects of plant growth and development. Y2H is advantageous for studying protein-protein interactions by enabling the screening of large protein libraries to identify new protein partners. For example, Liu et al. (2020) used Y2H to construct a cDNA library from grape leaves infected with *P. viticola*, the causal agent of downy mildew. They screened for the targets of pathogenic factors in host grapes, providing insight into the molecular mechanisms of *P. viticola* infection. In conclusion, Y2H is known for its user-friendly nature and its ability to detect protein-protein interactions. When coupled with a complementary technique (co-immunoprecipitation, BiFC, FRET), it becomes even more powerful in providing detailed insights into the biological processes under study.

#### 3.2.3 Mating-based split-ubiquitin system

The mbSUS system is a powerful tool for studying protein-protein interactions similar to Y2H system and is particularly used for membrane proteins. It involves splitting the ubiquitin protein into two parts: Cub (C-terminal ubiquitin) and Nub (N-terminal ubiquitin). One protein of interest is fused to Cub, while the other is fused to Nub. When these proteins interact, the two halves of ubiquitin reassemble, triggering the expression of a reporter gene. This system has been set up in *Arabidopsis* to study the interactome of K+ channels (Obrdlik et al., 2004), afterwards it has been successfully employed to study protein-protein interactions in various plant species such as grape, as exhaustively reviewed by Xing et al., 2016. In grapevine, mbSUS has effectively been used to investigate the interactions of two VpCDPKs that directly interact with VpMAPK3/6 and VpACS1/2, which promot the expression of many defense-associated genes in response to powdery mildew infection (Hu et al., 2021). While mbSUS is a robust tool, it is important to note that its limitations, including false positives and negatives, and the fact that the yeast environment may not perfectly mimic plant cell conditions. Furthermore, Y2H-like assays face significant challenges when studying cytoplasmic proteins that require nuclear translocation for their function (Cuadrado and Van Damme, 2024). To overcome these limitations, careful experimental design and validation of results are crucial. By combining mbSUS with other techniques, such as co-immunoprecipitation, BiFC or the cytoplasmic specific Cytotrap (Mohan et al., 2023), researchers can gain a more comprehensive understanding of protein-protein interactions in grapevine.

#### 3.2.4 Bimolecular fluorescence complementation

BiFC is a method used to detect protein-protein interactions in living cells. This assay is based on the reconstitution of a fluorescent protein *in vivo*, named reporter, that is truncated in two non-fluorescent halves. These halves are then fused to the proteins of interest (the bait and the prey). When these proteins interact, the two halves of the fluorescent protein reconstitute into a functional fluorescent protein, emitting fluorescence that can be detected using fluorescence microscopy. The inverted fluorescence microscope enables the detection and localization of the fluorescent signal within the cell. Moreover, the emitted fluorescence intensity corresponds proportionally to the strength of the interaction. Higher fluorescence levels signify close or direct interactions, while lower levels suggest interactions within a complex (Morell et al., 2008). BiFC assays have been employed in high-throughput screens to reveal new protein-protein interactions in yeast (Sung and Huh, 2007), plant (Peiró et al., 2014) and mammalian cells (Grau et al., 2017). Utsugi et al. (2015) used BiFC to confirm the interaction between HvTIP1-2 and HvTIP3 in onion. They showed that when HvTIP1-2, which has established water transport capability, interacts with HvTIP31, which does not exhibit such activity, they form heterotetramers that enhance water permeability. Xue et al. (2019) used BiFC to evidence a direct physical interaction between LYK1 and LYK4 in *A. thaliana.* BiFC offers several advantages, including its ability to provide real-time, spatially resolved information about interactions in living cells without the need for complex biochemical assays. The simplicity and adaptability of BiFC make it suitable for high-throughput screening applications, allowing researchers to analyze multiple protein interactions all together. It is also a valuable tool for the study of membrane protein interactions (García-Murria et al., 2020; Barriga et al., 2021; Duart et al., 2021).

### 3.3 *In silico* techniques

In silico methodologies are a useful tool to work around the difficulties associated with traditional *in vitro* or *in vivo* approaches and to bridge the gap between knowledge of TMPs structures and properties. In silico techniques can be applied on two diverse levels: directly on the protein sequence, to predict the most probable topology or the most favored 3-D structure, or directly to the structure (experimental or predicted). In this latter case, the in silico methodologies are employed principally to predict protein function or protein behavior; therefore, they make it possible to simulate protein-protein interactions, protein-ligand interactions or generally membrane system model behaviors. In the following paragraphs, a description of the principal methods applied for *in silico* predictions and of their applicability is presented.

#### 3.3.1 Topology and function prediction of transmembrane proteins

Prediction methods have been developed to provide valuable structural insights into TMPs, addressing their biological roles and the challenges associated with experimentally determining their three-dimensional structures. Topology prediction methods focus on two key aspects: the localization of transmembrane regions and their orientation. The hydrophobicity of buried amino acids is used to predict transmembrane regions, while the “positive-inside” rule aids in determining the orientation of these segments (Von Heijne, 1992). Various networks have been designed to predict TMPs, each employing different approaches. Some networks, such as TMpred and DAS, analyze local properties of amino acid sequences using a sliding window approach to detect sub-sequences that span the membrane. Others, like PHD, utilize a neural network and can also be classified as local methods. In contrast, global prediction methods such as TMHMM 2.0 and HMTOP employ Hidden Markov Models to determine the most statistically probable topology for the entire protein. Additionally, hybrid approaches, including MEMSAT 1.5, Toppred22, and TMAP, combine elements of both local and global prediction strategies (Möller et al., 2001). Before the advent of AI, several limitations in prediction techniques were identified (Tsirigos et al., 2018). However, recent advancements in AI-based approaches have increased the accuracy of these predictions. For instance, MemBrain 3.0 integrates a transmembrane helix prediction module with an orientation prediction module, utilizing a support vector machine classifier and an MMA strategy (Feng et al., 2020). These methods generally perform well for predicting alpha-helical or beta-barrel topologies but still struggle with non-standard topological features. Martinez-Navarro et al. (2024) analyzed full-length amino acid sequences using hybrid approaches to study the domain architecture of the TMP LecRLKs in *Vitis vinifera*. These methodologies are also applied to indirectly determine the cellular localization of gene products. For example, in a study on the LAR protein, which plays a key role in flavonoid synthesis in *A. thaliana*, the absence of a transmembrane region suggested a cytoplasmic localization of the protein (Han et al., 2018). To gain a deeper understanding of protein function, various computational and experimental approaches have been employed. Among these, the assessment of Gene Ontology (GO) terms has been extensively used to functional similarity between proteins. GO terms provide insights into a protein cellular localization, involvement in biological processes, and molecular functions. Since interacting proteins often share similar pathways, processes, functions, or cellular compartments these methods are particularly effective for categorizing interactions based on the similarity of their associated GO terms (Grassmann et al., 2024). Protein-protein interaction networks further provide valuable insights into the functional relationships between proteins of the same family and with other cellular proteins. For example, the probable functions of Rboh proteins in *Aquilaria* species were predicted on the basis of assignment of GO terms. These terms revealed interactions with various signaling components, such as receptor kinases, calcium sensors, and transcription factors (Begum et al., 2024). Overall, these prediction approaches offer several benefits, such as determining protein localization and orientation, along with accurate predictions for both alpha-helical and beta-barrel topologies. However, they are also prone to certain limitations, particularly the frequent misprediction of signal peptides in TMPs.

#### 3.3.2 AI-based prediction methods and deep learning

Shortly after the first experimental determinations of protein three-dimensional structures, computational methods were developed to predict protein folding based exclusively on amino acid sequences. Over the past 70 years, continuous advancements in these methods have been made, particularly with the integration of AI and deep learning techniques, which have led to significant innovations. These advancements have garnered recognition within the scientific community for producing predictions that closely resemble the quality of experimentally determined structures (Bordin et al., 2023). Among the leading deep learning methods for protein structural modelling are AlphaFold2 (Jumper et al., 2021) and ESMFold, a large language model for protein sequences (Lin et al., 2023). Nguyen et al. (2024) compared structures resolved through experimental methods with predictions obtained from these deep learning approaches. The findings indicate that high-confidence predictions are achieved in many regions of the proteins, while lower confidence is observed in areas lacking a defined structure and exhibiting significant flexibility, such as the connecting segments between TMPs. Specifically, the study evaluated the structural modeling of ion channels generated by these methods against experimental Cryo-EM structures. It was found that AlphaFold2 successfully predicted the majority of the hNaV1.8 domains with very high confidence scores (pLDDT > 90). In contrast, RoseTTAFold2 predicted most TMP regions with lower confidence (50 < pLDDT < 70) but accurately modeled the pore domain. Meanwhile, ESMFold demonstrated good confidence in predicting the voltage-sensing domains, pore domain, and extracellular loop regions (70 < pLDDT < 90). Currently, a search for protein models from *V. vinifera* in the AlphaFold database yields 183 modeled structures corresponding to sequences reviewed in SwissProt. In contrast, the Transmembrane Protein Structure Database (TmAlphaFold) (Dobson et al., 2023) provides 64 results, of which 51 exhibit excellent evaluations of their corresponding models. Overall, these methodologies bridge the gap between available sequences and resolved structures, enabling the prediction of protein 3D structures directly from amino acid sequences. While these methods offer several advantages, including the ability to simulate interaction formation and effectively build TMP systems, they also face limitations, such as the availability of 3D protein structures and challenges in predicting TMPs.

#### 3.3.3 Protein function prediction

Computational methods for studying proteins are invaluable for predicting three-dimensional structures and simulating protein-protein interactions, protein-ligand interactions, and molecular dynamics. Reliable molecular interaction simulations require that a protein’s three-dimensional structure be either experimentally determined or predicted with high confidence. Various docking software is available for investigating protein interactions, including GRAMM-X (Tovchigrechko and Vakser, 2006), ClusPro (Comeau et al., 2004), HDOCK (Yan et al., 2020), ZDOCK (Pierce et al., 2014), LZerD (Christoffer et al., 2021), and HADDOCK (Van Zundert et al., 2016). While these tools facilitate protein-protein docking, they typically do not account for the explicit flexibility of the modelled partners, although some can address this limitation through energy minimization. In silico methods are particularly useful for predicting membrane-associated protein assemblies. For instance, LightDock combines efficient rigid-body docking with artificial intelligence algorithms and employs flexible refinement using HADDOCK to resolve potential clashes at the interface (Roel-Touris et al., 2020). Other approaches, like JabberDock (Rudden and Degiacomi, 2021), utilize spatial and temporal influence density maps derived from short molecular dynamics simulations, while MPDock (Alford et al., 2015) leverages existing Rosetta sampling and scoring methods. A novel approach, AlphaFold-Multimer (Evans et al., 2021), is specifically trained for multimeric inputs with known stoichiometry, significantly improving the accuracy of predicted interfaces compared to the original single-chain AlphaFold. Selecting the most suitable method for protein interaction predictions is closely tied to the specific properties of the proteins being studied. The CAPRI competition, which stands for Critical Assessment of PRedicted Interactions, provides a valuable resource for guiding this choice by evaluating the success rates of various methods (Vakser, 2014). A notable example is Voss et al. (2023), who utilized AlphaFold-Multimer to predict the dimerization of EDS1 in Vitis vinifera. Their findings suggest that VvEDS1 dimers may serve as reservoirs for forming heterodimers with other proteins crucial for immune signaling in plants, demonstrating the significant impact of AI on understanding host-microbe interactions. Simulation tools offer several advantages, including tailored options for membrane-protein interactions and membrane remodeling (Goossens and De Winter, 2018). However, the lack of a uniform simulation method and the reliance on rigid body models can limit the accuracy of the resulting predictions.

## 4 Conclusion

Despite significant advancements in the identification and study of TMPs in plant growth, development, and stress responses, further research is necessary to fully understand their roles in grapevine defense against biotic stress. Existing studies have underscored the importance of specific TMPs such as LYKs, Rboh and SWEET in this context. However, the precise mechanisms underlying TMP-mediated intracellular signal transduction remain unclear, particularly in terms of how receptors transmit signals and the detailed pathways involved. Additionally, different TMPs involved in defense mechanisms have been identified in other plant species (see Zhou et al., 2022), and expanding research to them in grapevine could help uncover conserved mechanisms and contribute to a broader understanding of TMP functions in grape immunity. Emerging technologies present unique opportunities to resolve these gaps with greater accuracy. CRISPR/Cas9 technology, for instance, offers a promising, transgene-free method for precisely editing TMP-related genes, and has already been successfully applied in grapevine research. Furthermore, advanced molecular imaging techniques and high-resolution mass spectrometry can provide critical insights into the mechanisms of TMP action and interaction dynamics, shedding light on their functional roles within plant cells. The study of TMPs would also benefit from the integration of multi-omics approaches, such as proteomics and transcriptomics (such as single-cell RNASeq), to construct a more comprehensive understanding of the signaling pathways and interaction networks involved in biotic stress responses. As more TMPs are discovered, deeper exploration of their structural, regulatory, and functional diversity will be essential for advancing our understanding of their multifaceted roles in grape biology.
